# Insights into the Ecology and Evolutionary Success of Crocodilians Revealed through Bite-Force and Tooth-Pressure Experimentation

**DOI:** 10.1371/journal.pone.0031781

**Published:** 2012-03-14

**Authors:** Gregory M. Erickson, Paul M. Gignac, Scott J. Steppan, A. Kristopher Lappin, Kent A. Vliet, John D. Brueggen, Brian D. Inouye, David Kledzik, Grahame J. W. Webb

**Affiliations:** 1 Department of Biological Science, Florida State University, Tallahassee, Florida, United States of America; 2 Biological Sciences Department, California State Polytechnic University, Pomona, California, United States of America; 3 Department of Biology, University of Florida, Gainesville, Florida, United States of America; 4 St. Augustine Alligator Farm Zoological Park, St. Augustine, Florida, United States of America; 5 Wildlife Management International, Karama, and School of Environmental Research, Charles Darwin University, Darwin, Northern Territories, Australia; College of the Holy Cross, United States of America

## Abstract

**Background:**

Crocodilians have dominated predatory niches at the water-land interface for over 85 million years. Like their ancestors, living species show substantial variation in their jaw proportions, dental form and body size. These differences are often assumed to reflect anatomical specialization related to feeding and niche occupation, but quantified data are scant. How these factors relate to biomechanical performance during feeding and their relevance to crocodilian evolutionary success are not known.

**Methodology/Principal Findings:**

We measured adult bite forces and tooth pressures in all 23 extant crocodilian species and analyzed the results in ecological and phylogenetic contexts. We demonstrate that these reptiles generate the highest bite forces and tooth pressures known for any living animals. Bite forces strongly correlate with body size, and size changes are a major mechanism of feeding evolution in this group. Jaw shape demonstrates surprisingly little correlation to bite force and pressures. Bite forces can now be predicted in fossil crocodilians using the regression equations generated in this research.

**Conclusions/Significance:**

Critical to crocodilian long-term success was the evolution of a high bite-force generating musculo-skeletal architecture. Once achieved, the relative force capacities of this system went essentially unmodified throughout subsequent diversification. Rampant changes in body size and concurrent changes in bite force served as a mechanism to allow access to differing prey types and sizes. Further access to the diversity of near-shore prey was gained primarily through changes in tooth pressure via the evolution of dental form and distributions of the teeth within the jaws. Rostral proportions changed substantially throughout crocodilian evolution, but not in correspondence with bite forces. The biomechanical and ecological ramifications of such changes need further examination.

## Introduction

**Figure 1:**
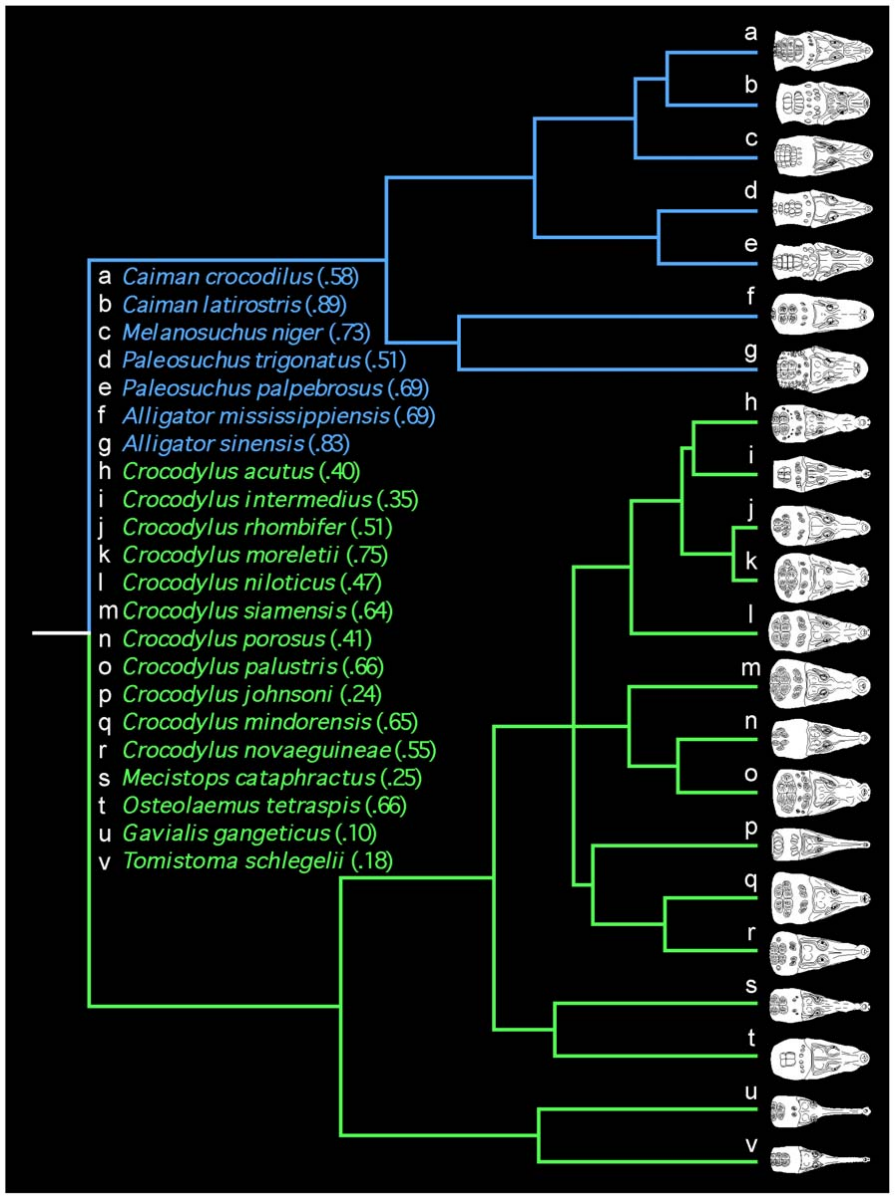
Phylogenetic hypothesis for extant Crocodylia showing variation in rostral proportions. The cladogram is based on reanalysis (see Materials and Methods) of molecular data from Gatesy and colleagues [2] using maximum likelihood and non-parametric rate-smoothing with branch lengths proportional to time. Lineages shown in blue represent caiman (a–e) and alligators (f,g) ( = Alligatoridae), and those in green crocodiles (h–t) and gharials (u,v) ( = Crocodylidae+Gavialidae). The Yacare caiman, *Caiman yacare* is not shown for it was not utilized in the Gatesy et al. [2] analysis. Dorsal views of heads are modified from Wermuth and Fuchs [53] and standardized to the same length to show relative differences in rostral form. Bracketed numbers following taxon names are the mean rostral proportions or RP ( = mid-rostral width/snout length) for each taxon from our study. Phylogenetic Independent Contrasts were performed on these 22 species; however, bite force, tooth pressure, and morphometric measurements and subsequent TIPs analyses were performed for all 23 extant taxa, including *Caiman yacare*.

**Figure 2:**
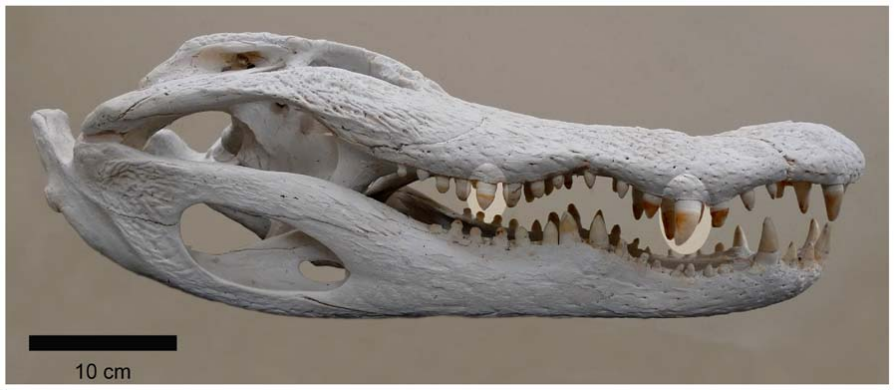
Skull and jaws of a wild adult American alligator (*Alligator mississippiensis*) showing the prominent teeth used for initially seizing and crushing prey. The most prominent caniniform and molariform teeth of the upper jaw that are associated with the convexities in the maxilla are highlighted. Because of their greater length relative to the adjacent teeth, and the propensity of crocodilians to bite unilaterally, the crowns of these teeth typically initiate contact with prey during biting. This specimen demonstrates the natural *in situ* condition of the teeth, which often fall out during skeletonization and must be reattached. As such the natural prominence of the teeth is sometimes not represented in prepared specimens. The caniniform teeth in crocodilians are longer, more slender, and generally have rounder cross-sectional shapes than the molariform teeth. In *A. mississippiensis* the apices of the caniniforms are fairly dull, whereas in more piscivorous species they are sharp and needle-like. Besides being utilized for seizing prey, caniniform teeth are also used in fighting, defense, aggression, and display. Crocodilian molariforms, on the other hand, are shorter and are typically blunter-tipped than the caniniform teeth. They range interspecifically from having a rounded bulbous morphology to being laterally compressed and blade-like. The intermediate condition seen here is characteristic of *A. mississippiensis*. Molariform teeth are primarily used for crushing and gripping prey in preparation for swallowing, but are also utilized for display and seizing prey.

Despite their large size (1.2–6.7 m total length [1]), crocodilians (Crocodylia: Alligatoridae: [alligators and caimans]; Crocodylidae: [crocodiles]; Gavialidae: [Indian and Malay (“false”) gharials]; [2], [3]) are remarkably stealthy predators – adept at stalking and ambushing prey in and around freshwater and estuarine environments. For the most part, their post-cranial anatomy related to locomotion is similar between species [4], [5]. Conversely adult body sizes and cranio-dental anatomy are conspicuously variable [3], [6] (Figure 1). Adults of all species are opportunistic feeders with diets that can include invertebrates, fish, snakes, turtles, birds and mammals [1], [7]. This is especially true of dietary generalists with teeth and snouts that occupy the middle ground among crocodilians with regard to sharpness and width, respectively. These include taxa such as the saltwater crocodile (*Crocodylus porosus*) and American alligator (*Alligator mississippiensis*) (Figures 1 and 2). On the other hand, those with extreme rostro-dental morphology tend to have more specialized diets. Several extremely slender-snouted forms with needle-like teeth, such as the Australian freshwater crocodile (*Crocodylus johnsoni*) and the Indian gharial (*Gavialis gangeticus*), consume a preponderance of small compliant prey such as fish, insects, and crustaceans [1], [7], [8] (Figure 1). Their elongated jaws, although structurally weak in bending [9]–[11], afford a broad strike zone during side-to-side head motions, rapid distal jaw closure, and we suspect, less obstructed vision when targeting prey. The broad-snouted caiman (*Caiman latirostris*) and Chinese alligator (*Alligator sinensis*) have blunt rostra and dull bulbous teeth for consuming hard-shelled mollusks [1], [7], [12] (Figure 1). This rostro-dental morphology helps to ensure enhanced structural rigidity through lower bending moments [9]. Additionally, high bite forces occur at all tooth positions due to their proximity to the jaw joint [9]–[11]. Finally, the dwarf caimans (*Paleosuchus trigonatus* and *Paleosuchus palpebrosus*) have dog-like vaulted rostra, and teeth with intermediate sharpness (Figure 1). Both feed at the water's edge; *Paleosuchus trigonatus* also forages on land for snakes, pacas and porcupines [1], [7], [13]. Their dorso-ventrally expanded snouts enhance rigidity in the plane of biting through increased area moments of inertia [9], [10].

The biomechanics of crocodilian feeding is poorly understood. Most notably it is not known how crocodilian bite forces and tooth pressures (bite force/tooth contact area) relate to rostro-dental and body size variance, dietary ecology, or evolutionary diversifications. Adult bite forces are only known for *Alligator mississippiensis* [14], [15], but are assumed to vary considerably among taxa. This is because of marked differences in the robustness of crocodilian teeth and jaws, dietary constituency (e.g. hard versus compliant prey [16]), and perhaps myology [17]–[19]. Recent computerized finite element modeling of crocodilian skulls supports this hypothesis [10] [11]. Bite forces are predicted to vary nine-fold among animals scaled to the same head size. Extremely low forces are posited in the delicate, slender-snouted forms and highest values in the robust, blunt-snouted taxa. The concurrent effects of interspecific differences in body size on bite force have not been explored, nor have the effects of phylogeny. Likewise, to our knowledge, tooth pressures (which reflect how such forces are actually transmitted to the prey) have not been studied in reptiles. (They are however known or estimated for humans, and a few animals such as sharks and other fish [20]–[22].)

Here we formally tested the longstanding hypothesis that crocodilian rostal proportions positively correlate with the capacity for bite-force generation. In addition, owing to the lack of speculation on how absolute bite forces and tooth pressures differ among extant crocodilians, we tested the hypothesis that these values scale isometrically with body mass.

We directly measured bite forces in sexually mature adults of all 23 extant crocodilian species [23] (Table 1) and inferred their peak tooth pressures at the prominent upper caniniform teeth (at the maxilla convexity near the front of the jaws) where prey are initially seized, and at the prominent upper molariform teeth (at the maxilla convexity nearer the back of the jaws) where food items are orally processed (Figure 2; also see Materials and Methods). We then tested the extent to which variation in forces and pressures could be explained by body size and rostral type. Spurious correlations were avoided by examining the effects of phylogenic relationships using independent contrasts. Finally, the biomechanical-performance traits were mapped onto a highly robust, re-estimated DNA sequence phylogeny to visualize character evolution and make evolutionary inferences about the role feeding biomechanics played in crocodilian ecological diversifications (see Materials and Methods).

**Table 1:** Anatomical measurements, and bite-force performance for extant Crocodylia.

Taxon	N	MRP	MBM	RBM	MTL	RTL	MBF	RMBF	MTFR	CBF	RCBF
**Crocodylidae**											
*Crocodylus acutus*	2	0.40	132	100–164	294	270–318	3999	3643–4355	1.54	2599	2368–2830
*Crocodylus intermedius*	1	0.35	182	182	340	340	6276	6276	1.59	4283	4283
*Crocodylus johnsoni*	5	0.24	20	7–43	167	134–215	1292	859–1863	2.05	629	418–856
*Crocodylus mindorensis*	1	0.65	69	69	244	244	2736	2736	—	—	—
*Crocodylus moreletii*	1	0.75	110	110	284	284	4399	4399	1.43	3069	3069
*Crocodylus niloticus*	2	0.47	86	86–87	250	240–261	3043	2914–3172	1.51	2007	1991–2023
*Crocodylus novaeguineae*	2	0.55	154	123–186	303	291–315	5360	4782–5938	1.36	3928	3547–4310
*Crocodylus palustris*	1	0.66	207	207	332	332	7295	7295	1.74	4194	4194
*Crocodylus porosus*	7	0.41	272	36–531	344	202–459	8983	1446–16414	1.57	5792	930–11216
*Crocodylus rhombifer*	3	0.51	52	30–65	214	187–246	2107	1392–3127	1.52	1379	917–2035
*Crocodylus siamensis*	3	0.64	69	40–87	238	212–263	3415	2073–4577	1.53	2227	1357–2891
*Mecistops cataphractus*	3	0.25	67	50–95	247	231–262	2082	1704–2447	—	—	—
*Osteolaemus tetraspis*	5	0.66	17	9–34	147	124–183	1787	1375–2509	1.53	1164	902–1588
**Gavialidae**											
*Gavialis gangeticus*	2	0.10	112	103–121	326	318–334	1895	1784–2006	2.06	924	819–1030
*Tomistoma schlegelii*	3	0.18	142	79–255	347	290–405	3397	1704–6450	1.62	2099	1052–3985
**Alligatoridae**											
*Alligator mississippiensis*	15	0.69	142	47–297	285	213–372	5117	2442–9452	1.54	3340	1414–6162
*Alligator sinensis*	4	0.83	14	12–15	150	140–155	1084	894–1357	1.48	735	555–963
*Caiman crocodilus*	4	0.58	20	18–25	166	166	1215	1148–1303	1.48	821	759–894
*Caiman latirostris*	5	0.89	30	16–45	167	157–177	1467	1050–2420	1.37	1063	777–1672
*Caiman yacare*	5	0.69	18	17–23	162	162	971	712–1192	1.50	646	485–900
*Melanosuchus niger*	3	0.73	59	31–103	246	304	2696	1779–4310	1.42	1911	1180–3112
*Paleosuchus palpebrosus*	3	0.69	13	12–14	133	133	900	667–1125	1.56	576	436–711
*Paleosuchus trigonatus*	3	0.51	22	19–28	150	143–156	1082	1058–1125	1.59	682	658–720
**Total**	**83**			**7–531**		**124–459**		**667–16414**			**436–11216**

**N** = Number of Specimens.

**MRP** = Mean Rostral Proportion (mid-rostral width/snout length).

**MBM** = Mean Body Mass (kg).

**RBM** = Range of Body Masses (kg).

**MTL** = Mean Total Length (cm).

**RTL** = Range of Total lengths (cm).

**MBF** = Taxon Representative Molariform Bite Force (N).

**RMBF** = Range of Molariform Bite Forces (N).

**MTFR** = Mean Tooth-Fulcrum Ratio (QA joint-Caniniform tooth/QA Joint-Molariform tooth).

**CBF** = Estimated Taxon Representative Caniniform Bite Force (N).

**RCBF** = Range of Estimated Caniniform Bite Forces (N).

## Results

**Figure 3:**
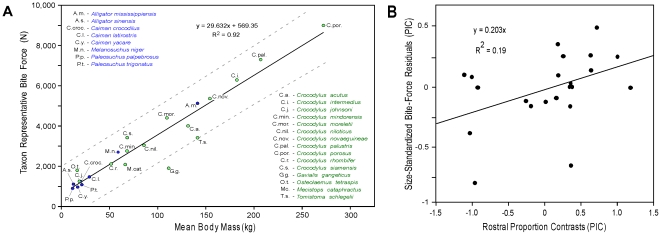
Taxon representative adult bite forces for extant Crocodylia with respect to mean body mass and the relationship between rostral proportion and force generation. (*A*) Members of the Alligatoridae are shown in blue, and members of the Crocodylidae+Gavialidae in green. The OLS regression equation describes the strong correspondence between body mass and bite force. Extant alligators and caiman (Alligatoridae), and crocodiles (Crocodylidae) show comparable relative bite-force capacities. Note that only *Gavialis gangeticus* is a statistical (i.e. outside the 95% confidence interval) low-force outlier. (*B*) Linear regression of the size-standardized residual bite force versus rostral proportion phylogenetic independent contrasts showing the low correlation between these after accounting for phylogeny and body mass.

The results of our study revealed taxon representative molariform bite forces ranging from 900 to 8,983 N (202 to 2,019 lbs) (*Paleosuchus palpebrosus* and *Crocodylus porosus* respectively; Table 1; Figure 3A). Body mass is the primary determinant of crocodilian force generation in both the raw data analysis (TIPs: R^2^ = 0.92) and phylogenetically corrected analysis (PIC: R^2^ = 0.87, p<0.0001). The reduced major axis (RMA) scaling coefficient for log-transformed taxon representative bite force regressed against log-transformed body mass was 0.708±0.111 (95% CI), which is not statistically different from isometry (scaling coefficient = 0.667). Only the forces for *Gavialis gangeticus* in the TIPS analysis are significantly atypical (lower) than those of extant Crocodylia as a whole (Figure 3A). Those for the Malay gharial (*Tomistoma schlegelii*) are moderately low. Interspecific differences in rostral proportions (Figure 1; Table 1) explain just 19% of the remaining variance in the size-standardized phylogenetically corrected data set (Figure 3B). This represents only 2.5% of the total variance from the aforementioned phylogenetically corrected analysis. Thus, the hypothesis that crocodilian rostal proportions positively correlate with bite-force capacity, while statistically significant (p = 0.03), is not supported as a major predictor of force even after correcting for size.

**Figure 4:**
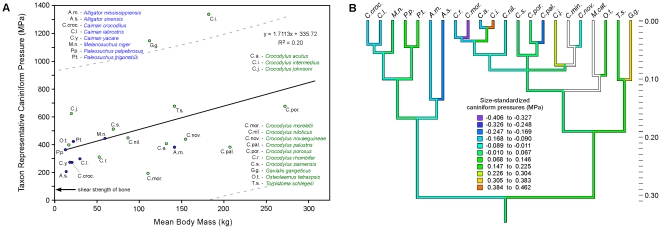
Caniniform pressure values for extant Crocodylia, their phylogenetic distribution, and inferred ancestral character states. (*A*) Members of the Alligatoridae are shown in blue, and members of the Crocodylidae+Gavialidae in green. The OLS regression equation describes the weak relationship between body mass and caniniform pressure. Note that slender-snouted piscivorous to semi-piscivorous ecomorphs (*Gavialis gangeticus and Crocodylus intermedius*, respectively) show exceptionally high-pressure values (outside the 95% confidence interval), and *Crocodylus johnsoni* shows pressures expected of animals nearly a magnitude in size larger. Other ecomorphs show much lower and similar relative values. The arrow indicates the typical ultimate shear strength of bone. (*B*) Ancestral character-state reconstruction using squared-change parsimony of size-standardized caniniform pressures. Residual caniniform pressure values are color coded to MPa (squared-change parsimony; squared length = 19.491). Vertical scale is in relative time, with the outgroup/ingroup root arbitrarily set to 1.0. High relative pressures were achieved independently in *Crocodylus intermedius*, *Gavialis gangeticus*, and *Crocodylus johnsoni*. Uncolored branches represent taxa for which the caniniform teeth were shed or broken, and so pressure estimation was not possible.

**Figure 5:**
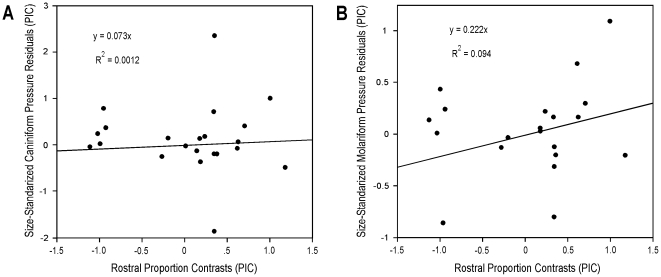
Linear regressions of residual caniniform tooth pressures, and residual molariform tooth pressures versus rostral proportion phylogenetic independent contrasts. The (*A*) residual caniniform, and (*B*) residual molariform regressions show the low correlation between these parameters after accounting for body mass and phylogenetic relatedness.

The taxon representative caniniform tooth-pressure values ranged from 195 to 1,344 MPa (28,282 to 194,931 psi) (Morelet's crocodile – *Crocodylus moreletii*, and the Orinoco crocodile – *Crocodylus intermedius*, respectively; Table 2, Figure 4A). These values also trend positively with increasing body mass, but are highly variable (TIPs: R^2^ = 0.20; PIC: R^2^ = 0.19, p = 0.09). The RMA scaling coefficient for log-transformed taxon representative caniniform tooth pressure regressed against log-transformed body mass was 0.490±0.203 (95% CI), which is greater (i.e. positively allometric) than isometry (scaling coefficient = 0.000), and so did not support our hypothesis. Exceptionally high values stand out in the slender-snouted, semi-piscivorous *Crocodylus intermedius*, and highly piscivorous *Gavialis gangeticus* (the latter generates the lowest relative bite force but also has exceptionally slender teeth with negligible contact area). The values for *Crocodylus johnsoni* are moderately high. All other ecomorph representatives show similar relative values. Size-standardized caniniform tooth pressures changed independently on multiple lineages (Figure 4B) and were uncorrelated with rostral proportions (PIC R^2^ = 0.001; Figure 5A).

**Table 2:** Dental measurements and pressure generation for extant Crocodylia.

Taxon	N	CCA	RCCA	CP	RCP	MCA	RMCA	MP	RMP
**Crocodylidae**									
*Crocodylus acutus*	2	5.78	5.78	410	410	5.50	4.93–6.08	728	716–740
*Crocodylus intermedius*	1	3.19	3.19	1344	1344	4.52	4.52	1388	1388
*Crocodylus johnsoni*	5	1.01	0.52–1.80	624	381–1078	1.56	0.51–2.14	832	565–1871
*Crocodylus mindorensis*	1	—	—	—	—	8.02	8.02	341	341
*Crocodylus moreletii*	1	15.72	15.72	195	195	14.24	14.24	309	309
*Crocodylus niloticus*	2	6.18	2.89–9.48	451	213–689	5.41	5.06–5.77	566	505–627
*Crocodylus novaeguineae*	2	9.30	6.99–11.61	439	371–508	5.04	4.91–5.17	1061	973–1149
*Crocodylus palustris*	1	10.97	10.97	382	382	14.42	14.42	506	506
*Crocodylus porosus*	7	8.33	3.98–9.57	679	234–1343	7.44	2.22–16.87	1207	300–2473
*Crocodylus rhombifer*	3	4.55	3.08–7.10	312	263–385	4.48	2.93–6.18	466	414–506
*Crocodylus siamensis*	3	6.59	3.32–9.85	513	293–732	5.52	4.73–6.67	616	402–760
*Mecistops cataphractus*	3	—	—	—	—	4.19	4.19	406	406
*Osteolaemus tetraspis*	5	2.94	1.37–5.07	400	241–660	8.79	4.73–13.91	203	139–343
**Gavialidae**									
*Gavialis gangeticus*	2	0.81	0.76–0.86	1154	958–1349	2.98	1.41–4.56	855	440–1270
*Tomistoma schlegelii*	3	2.99	1.72–1.84	678	613–737	4.28	3.14–5.05	790	337–1384
**Alligatoridae**									
*Alligator mississippiensis*	15	8.42	4.16–14.67	383	209–722	6.43	4.18–8.16	806	299–1568
*Alligator sinensis*	4	3.60	2.67–4.59	207	153–258	5.34	3.81–6.97	211	150–278
*Caiman crocodilus*	4	3.04	2.52–3.53	275	228–329	3.58	2.60–4.40	351	296–455
*Caiman latirostris*	5	3.99	2.21–6.47	298	173–487	4.18	2.43–6.79	372	226–489
*Caiman yacare*	5	2.60	1.56–4.23	276	164–387	3.46	1.76–5.92	319	185–515
*Melanosuchus niger*	3	4.15	3.04–5.71	446	320–545	6.55	3.92–9.22	417	274–509
*Paleosuchus palpebrosus*	3	1.58	1.25–2.01	365	350–393	2.18	1.15–3.63	493	309–793
*Paleosuchus trigonatus*	3	2.24	0.88–3.08	424	216–815	3.91	3.05–4.64	287	228–369
**Total**	**83**		**0.52–15.72**		**153–1349**		**0.51–16.87**		**139–2473**

**N** = Number of Specimens.

**CCA** = Taxon Representative Caniniform Contact Area @1 mm depth (mm^2^).

**RCCA** = Range of Caniniform Contact Areas @ 1 mm depth (mm^2^).

**CP** = Taxon Representative Caniniform Pressure (MPa).

**RCP** = Range of Caniniform Pressures (MPa).

**MCA** = Taxon Representative Molariform Contact Area @ 1 mm depth (mm^2^).

**RMCA** = Range of Molariform Contact Areas @ 1 mm depth (mm^2^).

**MP** = Taxon Representative Molariform Pressure (MPa).

**RMP** = Range of Molariform Pressures (MPa).

**Figure 6:**
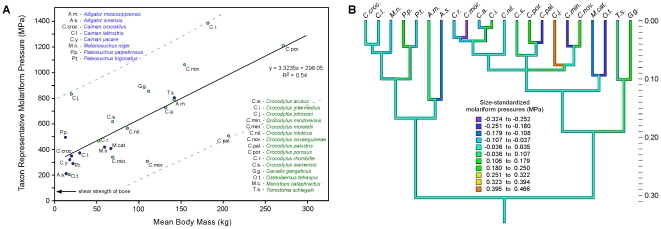
Molariform pressure values for extant Crocodylia, their phylogenetic distribution, and inferred ancestral character states. (*A*) Members of the Alligatoridae are shown in blue, and members of the Crocodylidae+Gavialidae in green. The OLS regression equation describes the relationship between body size and molariform pressure. Note that the range of values shows similar interspecific correspondence to the caniniform pressure data shown in Figure 4A. The arrow indicates the typical ultimate shear strength of bone. (*B*) Ancestral-state reconstruction using squared-change parsimony of size-standardized molariform pressures. Pressures are color coded to MPa. Vertical scale is in relative time, with the outgroup/ingroup root arbitrarily set to 1.0. The notable similarities between unrelated taxa and differences between related taxa illustrate the large amount of convergence for this trait among crocodilians.

Taxon representative molariform tooth-pressure values ranged from 203 to 1,388 MPa (29,443 to 201,312 psi) (Dwarf crocodile – *Osteolaemus tetraspis*, and *Crocodylus intermedius*, respectively; Table 2, Figure 6A). These are more strongly correlated with body mass than the caniniform data (TIPs: R^2^ = 0.54; PIC: R^2^ = 0.293, p = 0.008). The RMA scaling coefficient for log-transformed taxon representative molariform tooth pressures regressed against log-transformed body mass was 0.553±0.180 (95% CI), which is greater (i.e. positively allometric) than isometry (scaling coefficient = 0.000), and therefore did not support our hypothesis. None of the molariform pressure values are statistical outliers. Nevertheless, those for the slender-snouted *Crocodylus johnsoni* and *Crocodylus intermedius* are relatively high, and those for the broader-snouted generalists, the mugger (*Crocodylus palustris*), and Morelet's crocodile (*Crocodylus moreletii*) are relatively low. Pressures for all other ecomorphs, including *Gavialis gangeticus*, are comparable. Size-standardized molariform tooth pressures changed repeatedly in the phylogeny (Figure 6B) and were not significantly correlated with rostral proportions (PIC R^2^ = 0.094; Figure 5B).

## Discussion

The results of our investigation into the biomechanics and evolutionary ecology of crocodilian feeding revealed a number of unexpected findings. We found negligible support for the commonly held view that bite forces correlate strongly with rostral form – a proxy for strength. Rather, bite forces vary independently of rostral morphology, so much so that in some cases same-sized taxa from the extreme bounds of crocodilian rostal morphology and dietary ecology (e.g. the slender-snouted, *Crocodylus johnsoni* and robust-snouted, *Caiman latirostris*; Figure 1) show “pound for pound” comparable molariform bite forces (Figure 3A). During cladogenic events, when rostral form was modified into different types (presumably to allow access to different prey), bite forces were just as likely to increase as decrease.

Our findings suggest that for crocodilians of similar body mass, the same absolute bite forces will be generated at equal distances from the quadrate-articular joint. A consequence of this is that more slender-snouted forms will at the same time experience higher stresses to their jaws since they have lower area moments of inertia with which to resist bending. Furthermore, since they have relatively longer snouts, equal loads applied at the tip of the jaws will lead to higher absolute bending moments than in shorter-snouted forms. This begs the question: How do slender-snouted species sustain bite forces typical of more robust-snouted crocodilians? We suspect the answer lies primarily in their prey selection. They target small prey relative to their size (e.g. fish and crustaceans, and/or birds and small mammals by the larger species) whose low inertia contributes little to resistance forces. It is also plausible that their jaws experience stresses closer to rupture strength (i.e. lower safety factor [24]) during feeding than the other ecomorphs. This is certainly the case during other behaviors such as fighting and defense, where they show a much greater propensity to sustain broken jaws [25].

Body size actually accounts for nearly all interspecific variance in adult crocodilian bite-force capacity, and these forces scale isometrically to body mass. Clearly a major factor in the evolutionary success of crocodilians stems from their long-term retention of a cranial musculoskeletal system that can generate sufficient force to procure and process near-shore prey across a broad range of body sizes. Only in the extremely slender-snouted *Gavialis gangeticus*, arguably the only truly piscivorous species, is there evidence of significant departure in performance, and this is reflected in their anatomy. These low-force biters independently evolved extremely hypertrophied, low-mechanical advantage adductor mandibulae muscles, and small, fusiform-fibered posterior pterygoid muscles that presumably accentuate rapid jaw closure [17], [19]. This enhanced jaw-closing performance was likely afforded at the cost of diminished bite-force capacity, which is consistent with our empirical findings for both molariform and estimated caniniform bite forces in *Gavialis gangeticus*.

The retention of relative bite-force capacity among crocodilians makes it apparent that the remarkably high bite forces first documented in adult *Alligator mississippiensis* [14], [15] are typical of most comparable-sized species, regardless of rostro-dental anatomy or diet. Even higher forces are to be found in larger species like the slender-snouted, semi-piscivorous *Crocodylus intermedius* and the medium-snouted generalist *Crocodylus porosus* – the largest extant taxon. (Our datum for one *Crocodylus porosus* individual, 16,414 N [3,689 lbs] represents the highest bite force measured in any animal. This value eclipses the highest recorded value in carnivoran mammals, 4,500 N [1,011 lbs] in the spotted hyena – *Crocuta crocuta* [26].)

Crocodilian bite-force retention can be used to predict forces in other specimens and species, including taxa known only from fossils (see Materials and Methods). For instance, scientifically documented 6.7-meter long *Crocodylus porosus* individuals [1] were likely capable of molariform bite forces of approximately 27,531 N to 34,424 N (6,187 to 7,736 lbs). In addition the historical range of adult bite-force values for Crocodylia as a whole may have spanned from 628 N to 102,803 N (141 to 23,102 lbs; in extinct 0.8 m TL, 1.99 kg *Procaimanoidea kayi* [27] and 11 m TL, 3,450 kg *Deinosuchus riograndensis* [28], respectively; see Materials and Methods).

No previous hypotheses exist regarding tooth pressures in crocodilians. Thus, the data we report provide new insights into how bite forces are conveyed through the most prominent teeth to allow these animals to seize prey, and initially puncture or drive cracks through their tissues. We found that both caniniform and molariform pressures scaled with positive allometry versus the expected isometric scaling value of 0.000. Notably the absolute pressures at both tooth positions were remarkably high. Values for all taxa exceeded the highest reported previously (147 MPa [21,321 psi]) for the giant extinct placoderm fish *Dunkleosteus* [22]), and pressures for some individuals were as much as 17-fold higher (Table 2). In addition we discovered that the caniniform and molariform teeth showed similar peak pressure values within individuals and species (Table 2). This occurred despite differing shapes and functions relative to one another (Figure 2) and unequal bite forces (Table 1). (The caniniform forces are 36% lower on average because they are further from the quadrate-articular joint fulcrum; Table 1.) We suspect the reason for the similarity is that both tooth types are composed of the same dental constituents (enamel and von Ebner's dentine) and must be able to damage, and yet sustain impacts with the same tissue types when feeding. Notably, the pressure values in all taxa considerably exceed the ultimate shear strength of bone (65–71 MPa; Figures 4 and 6), the strongest of the hard constituents (incl. dentine, enamel, calcium carbonate) they encounter in their potential prey [29]. This holds true even during the seizing of prey underwater where initial tooth pressures could be less since jaw-closing velocity diminishes by up to two-fold from pressure and frictional drag (see Materials and Methods). Clearly this biomechanical capacity is integral to the dietary plasticity of all living crocodilians. It was also certainly vital to the occupation of near-shore habitats by crocodilians over millennia – although prey types changed, the materials of which they were composed did not (e.g. [30]).

Crocodilian tooth pressures show negligible correlation with phylogeny (low K values, significant deviation from a Brownian motion model). This result suggests that convergent adaptation is contributing more signal than phylogenetic relatedness. Presumably, evolutionary changes that allowed dietary niche occupations were responsible for much of the variation. Nevertheless, ecomorph-specific tooth-pressure values are ambiguous. Only highly piscivorous *Gavialis gangeticus* and semi-piscivorous *Crocodylus intermedius* and *Crocodylus johnsoni* [1], stand out with respect to caniniform pressure generation in showing relatively high values (Figure 4A). (This is remarkable in the cases of *Gavialis gangeticus* and *Crocodylus johnsoni*. Their most prominent caniniform teeth are located more rostrally than in all other crocodilians where bite forces are relatively low; Table 1. Furthermore, *Gavialis gangeticus* generates the lowest relative bite forces among living crocodilians; Figure 3.) All other crocodilian ecomorphs (molluscivores, terrestrial foragers, broad-snouted generalists, and the slender-snouted generalists – such as *Tomistoma schlegelii* and the American crocodile – *Crocodylus acutus* [1], [7]) show similar values to each other that are relatively lower. What unite these ecomorphs are caniniform teeth that abruptly broaden – moving from the crown apex to the tooth neck. Should substantial, hard constituents be impacted during biting, or off-axis forces experienced, this tooth morphology provides for structural rigidity through reduced bending moments and increased area moments of inertia. However, this is afforded at the cost of rapidly diminishing tooth pressure following initial contact [31]. Conversely, the slender caniniform teeth of the piscivorous and semi-piscivorous ecomorphs ensure that less force is required to drive the teeth through prey. However, higher bending moments and low area moments of inertia put their long, narrow tooth crowns at risk of breakage. Tooth failure is presumably circumvented to some degree through the selection of prey with negligible hard tissues and low inertia (see above).

Molariform tooth-pressure values vary widely among crocodilians. For example the data for the similar-sized durophagous *Alligator sinensis* and *Caiman latirostris* span much of the range for other same-sized crocodilians (Figure 6A). No values are statistical outliers, and no definitive ecomorphological groupings exist. As mentioned above, crocodilian molariform pressures are comparable to those in the caniniform teeth. However, the bite forces at the molariform tooth positions are much higher since they are closer to the jaw's fulcrum. Because the molariform teeth are stouter, they are well suited for enduring higher resistance forces while at the same time generating pressures that, like the caniniform teeth, are initially sufficient to damage the hard constituents in their prey. Catastrophic failure of the prey's tissues is subsequently induced either by driving cracks [21] at the point of tooth engagement (the damage being more expansive in the stoutest-toothed forms), or by causing structural failure away from the point(s) of tooth engagement due to bite force alone.

The biomechanics behind the crocodilians' remarkable and long-term occupation of niches near the water-land interface are for the first time revealed. The breadth of our findings allows us to propose an integrative model that explains the evolution of ecologically relevant phenotypic traits. Body size, and not rostral proportions, explains nearly all interspecific differences in bite-force generation. The crocodilian musculo-cranial design allows for the generation of prodigious bite forces across a broad range of sizes (*Gavialis gangeticus* is the exception; see above). This suggests that scaling mediated change in size was a primary means by which these animals gained access to new feeding resources. The rampant size changes that occurred throughout crocodilian evolution in the fossil record [3], [6], [32] are testament to the importance of scale-mediated changes in the feeding biomechanics of these animals.

Changes in tooth morphology also facilitated shifts in crocodilians' diets. Tooth size and shape (i.e. cross-sectional area) dictate contact areas, which act in concert with bite forces to generate pressures. These determine performance with respect to the structure and mechanical properties of prey. Our results demonstrate that tooth pressures and snout morphology change independently of each other. We found evidence in support of ecomorphic specific performance in more piscivorous species with regard to initial tooth pressures. Others certainly exist, especially among durophagous species, but initial tooth pressures alone are insufficient to single out their biomechanical import. Our conceptual model leaves rostral shape, which is obviously very important with regard to the crocodilian diversification, to be explained more fully by its relevance to the positioning and numbers of teeth, jaw hydrodynamics, and resistance to torsion or bending during prey capture and processing [11]. Collectively, the data and methods from this study provide the quantitative biomechanical foundation for further exploration (particularly in fossil taxa) of the remarkable evolutionary success of these long-term predatory denizens of the water-land interface.

## Materials and Methods

### Data Collection

This study was carried out in strict accordance with the recommendations in the Guide for the Care and Use of Laboratory Animals of the National Institutes of Health. The research protocol was approved by the Animal Care and Use Committee of The Florida State University (Permit Number: 0011). The animals were manually secured and strapped down to a testing platform prior to bite-force experimentation, and all efforts were made to minimize suffering. No animals were injured during the execution of this research.

We tested all available sexually mature adult crocodilian specimens from research, conservation, and display specimens housed at the St. Augustine Alligator Farm Zoological Park, St. Augustine Florida, USA and Crocodylus Park, Darwin, AUS (Table 1). In total 83 adult (sexually mature) specimens representing all 23 extant crocodilian species currently recognized by the IUCN-SSC (Species Survival Commission of the International Union for Conservation) Crocodile Specialist Group ([23]; size range 1.24–4.59 m, 7–531 kg) were accessed. Multiple individuals were studied for 19 species (Table 1). Our analysis included both male and female specimens since prior studies on wild and captive *Alligator mississippiensis* bite forces revealed statistically indistinguishable performance in same-sized individuals (i.e. body mass, SVL, TL) [14], [15]. The results from the present study confirmed these findings (data not presented).

The bite forces were recorded using sandwich transducers and a portable charge amplification system specifically designed for use on crocodilians [14]. Two preliminary studies on growth series of captive and wild *Alligator mississippiensis* using this system showed that specimens consistently bit at values near the yield point of the dentition (safety factor = 1.0–1.4) and hence near maximal structural capacity (Note: ∼10% of wild *Alligator mississippiensis* teeth are fractured during normal usage prior to shedding; [33]), and bite-force values for captive specimens can be used to accurately model those in wild individuals when standardized to body mass [15]. Three to five bites were recorded for each animal, the highest of which was used in post-hoc analyses.

Forces were measured on land with the transducer centered below either the left or right most prominent maxillary molariform tooth (located at the maxilla convexity nearer the back of the jaws; Figure 2). This is an ecologically relevant location since it is where these animals primarily crush prey. Crocodilians stereotypically seize prey contacted by the teeth and jaws as the head is swiped to the side. They also process food on one side of the jaw. Thus, unilateral rather than bilateral tooth engagement best mimics natural feeding behavior. Additionally, unilateral crushing of prey at the molariform teeth commonly occurs with the head out of water in all species. Similarly, the seizing of prey using the caniniform teeth often occurs with the head out of water. Fortuitously the prominent molariform tooth position is at a comparable relative distance from the fulcrum across taxa, as an RMA plot of log-transformed fulcrum to molariform distance regressed against log-transformed body mass showed a scaling coefficient of 0.342±0.029 (95% CI), which is not different than isometry at 0.333. Therefore it provided a useful biomechanical standard of comparison in our testing. We took into consideration the effects of drag on force (and pressure generation; see below) during underwater feeding. Maximal velocity differences during terrestrial versus aquatic biting are no more than two-fold intraspecifically regardless of rostral form [31]. (Note: The effective bite force applied during sub-aquatic or terrestrial clenching bites [i.e. where the bite-force transducer or prey has already been seized and a new bite initiated] would be unaffected by drag. Our data show that the forces generated during such bites are at least 90% of the maximum values recorded during initial, defensive bites [14], [15], [31].)

Standard measures of size and morphometrics pertinent to feeding biology were then recorded (Table 1). These included body mass (BM), total length (TL), and rostral proportion (RP,  = mid-rostral width/snout length [measured midway between the anterior borders of the orbits to the tip of the rostrum]). In addition, dental putty molds (Knead-A-Mold; Townsend Atelier Inc., Chattanooga, TN) were made for the most prominent caniniform tooth, the primary tooth used to initially contact and seize prey, as well as for the most prominent molariform tooth used in the crushing of prey (Figure 2). These prominent teeth reside in alveoli at the apex of the maxilla convexities. They primarily act in isolation to initiate contact with the prey during seizing or crushing feeding behaviors. Their initial biomechanical performance can be directly linked to morphology and/or dietary ecology before the adjacent teeth become engaged as the tooth descends into prey or the padded transducer. (Note: the caniniform teeth in particular are also employed in defense and aggression where the same biomechanical performance measures studied here are also pertinent). The most pristine of any tooth pair was molded. Specimens for which both teeth were heavily damaged were not used in our analysis. Epoxy casts were made from the molds for use in post-hoc interspecific comparisons of absolute initial maximal tooth pressures.

The casts were indented normally to a depth of one mm in modeling clay. (We found that measurements <5% crown height were imprecise for the teeth of small taxa. Because of this we opted to use the minimal depth for which repeatable measurements of area could be made for all specimens.) The indentations were digitally photographed and the realized contact area normal to the direction of loading determined using NIH Image software (ImageJ64 v.1.42q, National Institute of Health, Bethesda, MD, USA). Initial molariform tooth pressures were determined by dividing the one mm contact area for each individual's molariform tooth into its respective molariform bite-force value. Initial caniniform pressure estimates were determined by dividing the one mm contact area for each individual's caniniform tooth into its respective estimated bite-force value. We analyzed the significance of the tooth-pressure values with respect to the shear strengths of the hardest constituents found in crocodilian prey. (Note: shearing is the primary means of failure for hard materials in biological systems [29].)

### Raw Data (“TIPS”) Analyses

To establish the effects of body size and morphology on bite force, we established a taxon representative value of bite force at the most prominent molariform tooth for each of the 23 crocodilian species (Table 1). For most taxa (n = 19) this was simply established using the mean maximal bite force with respect to mean body mass. These included species for which: 1) just two or fewer individuals were available for testing, or 2) species for which no statistical difference between representative values were found using either an ordinary least squares (OLS) linear-fit, or a power-law modeling of the data with body mass as the independent variable. Notably we found these to be taxa whose range of sizes in our sample was less than 70% of the size for the largest individual. However, for some taxa (i.e. *Alligator mississippiensis*, *Crocodylus johnsoni*, *Crocodylus porosus*, and *Osteolaemus tetraspis*—species for which the range of intraspecific body sizes in our sample was greater than 70% of the largest individual's size) it was necessary to account for scaling effects on bite force using power-law modeling. A moment calculation was used to infer the biting force simultaneously developed at the prominent caniniform tooth of each individual [34] (Table 1). Taxon representative caniniform bite forces were derived using the same protocol discussed above for the molariform bite-force data (see above). For comparing taxon representative bite forces, making extrapolations to fossil taxa, and deriving residuals for subsequent analyses, OLS regressions were used.

As with bite forces, we also established taxon representative values for caniniform and molariform contact area (Table 2). For most taxa (n = 19), this was done by averaging the one mm mean contact area data for each species (see above). For the same four taxa in which the range of intraspecific body size was greater than 70% of the largest individual's size (see above), we accounted for scaling effects on contact area using power-law modeling. Taxon representative molariform and caniniform tooth pressures were then derived using the same protocol discussed previously (see above) for taxon representative bite forces and contact areas.

Scaling relationships for taxon representative bite forces, as well as the taxon representative caniniform and molariform pressures, were determined using reduced major axis regressions to account for error in both the X and Y-variables, which were log-transformed. A best-fit regression and 95% confidence intervals were then constructed. Plots for which the scaling coefficient fell outside the confidence intervals of the best-fit regression were considered to be allometric. (Note: isometry for taxon representative bite forces regressed against body mass has a scaling coefficient of 0.667. This is because force increases as a square [i.e. as a function of muscle physiological cross-sectional area] while body mass, a volumetric measure, increases as a cube. On the other hand, isometric scaling of tooth pressures has a scaling coefficient of 0.000. This is because both the force and contact area parameters used to calculate tooth pressure increase as a square with respect to increases in body mass. Thus, tooth pressure is expected to remain unchanged with respect to interspecific increases in body mass among crocodilians.)

After regressing the taxon representative bite forces against mean body mass for all species using OLS, we used the bite-force residuals to analyze the effects of rostral proportions on relative bite force independent of size for each taxon. While these results are useful for visualizing the relationships between size, morphology, and bite force, they do not take into account the phylogenetic relationships among taxa. In order to provide a visual heuristic of the pattern of evolution, all TIPs data were mapped onto the phylogeny described below under maximum likelihood using Mesquite 2.5 [35]. In order to account for correlations due to shared history and gain statistical rigor, we proceeded to investigate phylogenetic independent contrasts in the manner described below.

### Phylogeny Reconstruction

We chose to use the exceptionally comprehensive gene database from Gatesy et al. [2] to explore the effects of phylogeny on the character evolution of crocodilian feeding biomechanics. We acknowledge that, as with nearly all species rich phylogenies, competing hypotheses exist. Among published molecular studies some sub-clade relationships are debated (e.g. *Crocodylus porosus, and Crocodylus acutus* [36]–[39]). Nonetheless, the comprehensive sampling of genetic data as in Gatesy et al. [2] has yet to occur to enable more rigorous comparisons across all taxa. Morphologically derived trees provide consistent hypotheses with regard to the position of *Gavialis gangeticus* as outside of Crocodyloidea [3], [6], and some morphology-only [6], [40] and combined molecular and morphology analyses [41] support the African slender-snouted crocodile (*Mecistops cataphractus*) as sister to *Crocodylus* instead of *Osteolaemus tetraspis* [6]. Regardless, we are confident the tree used here is the best available estimate of the true phylogeny that also contains time-correlated branch lengths (necessary for PIC analysis). Character mapping and exploring the potential evolutionary and ecological ramifications of our data using other competing hypotheses is beyond the scope of the present study, but will be the topic of future analyses.

For our study, an aligned DNA sequence matrix was obtained from John Gatesy (University of California, Santa Barbara), consisting of published sequences for the nuclear genes RAG-1, BDNF, ATP7A, LDHa, c-myc, c-mos, DMP1, ODC, and 18S/28S rflp, and portions of the mitochondrial genes nd6, cyt b, the intervening glutamine tRNA, control region, 12S, and 16S [2]. Because the published trees did not include branch lengths, and were not ultrametric (i.e. proportional to time), we re-estimated the phylogeny. Some species were not represented for all genes. Most notable was the New Guinea crocodile (*Crocodylus novaeguineae*) that included only the ND6/cyt b and 18S/28S regions. No sequence data were available for *Caiman yacare*, and so it was not included in the phylogeny, nor in subsequent phylogenetic independent contrasts (PIC). (Note: it was, however, included in analyses of the raw [TIPs] data.) We followed Gatesy *et al.* [2] in designating Paleognathae and Neognathae as outgroups in all phylogenetic analyses. However these were pruned from the tree prior to conducting the PIC analyses. Alignments were checked by eye. Small modifications were made to maintain codon integrity in reference to the translated amino acid sequence using MacClade [42], but sequences otherwise conformed to the published alignments. A maximum likelihood (ML) search was conducted using PAUP [43] under the GTR+I+G model as indicated by Modeltest [44] and the Akaike Information Criterion. Parameters were estimated from a randomly chosen tree among the most parsimonious trees found under equal weighting parsimony. Starting trees included the set of most parsimonious trees in addition to 10 random addition sequence replicates. All searches found the same single tree (Figure 1), congruent with the slightly less resolved tree in Gatesy et al. [2], and identical to that in the more recent Gatesy and Amato [45] analysis with the exception of our tree resolving one trichotomy near the tip in *Crocodylus*. The ML tree was made ultrametric using penalized likelihood in r8s [46], [47] and the ML branch lengths. Cross validation was conducted on the ML tree using a range of smoothing parameters from 1 to 1000. A smoothing parameter value of 3.2 was found to minimize deviations and was the value used in the final analysis.

### Phylogenetic Independent Contrasts

All morphological and mechanical variables (see Tables 1 and 2) were log-transformed with the exception of rostral proportions (RP). Transformations were done to normalize these data, which spanned a large, 21-fold size range in mean body mass (Table 1). Because rostral proportions are a ratio, and therefore are already normalized to body size, log-transformation was unnecessary. These proportions were not significantly correlated with size, unlike all other variables. Phylogenetic signal was estimated by the K statistic using the picante package [48] in R [49]. K statistics ranged from 0.347 to 0.838. A value of 1.0 indicates these data are fit by a Brownian motion model, whereas values close to 0 indicate closely related taxa are less similar than expected under Brownian motion, as might be caused by adaptation or measurement error [50]. All variables exhibited significant phylogenetic signal (K>0.5) except log-molariform contact area and the log-pressure variables (K = 0.347–0.443; p = 0.209–0.344). Caniniform and molariform pressures were mapped onto the phylogeny using the Mk1 model for likelihood in Mesquite 2.5 [35]. PIC analyses were conducted using the ultrametric tree with the PDAP module [51] in Mesquite 2.5 [35]. The contrasts were standardized through division with their standard deviations (square-root of summed branch lengths). This effectively converted them to evolutionary rates. PDAP diagnostics (standardized contrasts regressed against their standard deviations) showed that only log-molariform contact area and log-caniniform pressure deviated significantly from a Brownian motion model (p<0.05). Because the tree contained one trichotomy, the degrees of freedom for the diagnostics were reduced by one. Since bite force was strongly correlated with body mass for both raw data (R^2^ = 0.92) and phylogenetically corrected log-transformed data (R^2^ = 0.87), we first created size-standardized variables. This was achieved by regressing contrasts of mean rostral proportions as well as log-transformed taxon representative bite forces, tooth contact areas, and tooth pressures against contrasts of log-mass and saved the residuals. To remove the effects of tooth size, the size-standardized residuals for pressure were also regressed against the size-standardized residuals of contact area. These size- and contact area-standardized residuals were saved. This effectively removed the evolutionary variance in pressure associated with changes in body mass and tooth cross-sectional area. These residuals for performance measures were then regressed against the rostral proportions. All regressions on PICs were constrained to pass through the origin. Throughout, we report TIPs results for ease of visualization, but due to non-independence of the raw data, statistical significance is reported only for PICs.

### Estimations of Bite Forces in Fossil Crocodilians and Large Extant Individuals

Our range of bite-force estimates for 6.7 m specimens of *Crocodylus porosus* was based upon the interspecific regression of mean body mass versus mean bite force (Y_(force, N)_ = 29.632x_(body mass, kg)_+569.35; R^2^ = 0.92; see Figure 3A) with an estimated mass of 1,308 kg from the intraspecific regressions of wild *Crocodylus porosus* from Webb and Messel [52]. A second bite-force estimate was acquired using an intraspecific regression for a captive growth series of this taxon (range = 0.96–531 kg; [31]) where y_(bite force, N)_ = 115.39x_(body mass, kg)_^0.7629^, R^2^ = 0.98). Note: Our previous research has shown that bite-force generation is statistically indistinguishable between same-sized (i.e. body mass, SVL, or TL) captive and wild *Alligator mississippiensis* [14], [15].

Our estimates of the upper and lower historical bounds of adult crocodilian bite forces were based on the interspecific regression of mean mass versus mean bite force (Y_(force, N)_ = 29.632x_(mass, kg)_+569.35; R^2^ = 0.92; see Figure 3A) with an estimated mean mass from our interspecific regression of captive crocodilians reduced by 25% [14], [15] to account for the lesser mean body mass of wild individuals compared to captives of equal TL. We used the 0.8 m TL *Procaimanoidea kayi* [27] to represent the lowermost bound of known size for Crocodylia. The 11 m TL *Deinosuchus riograndensis* [28] was used to represent the upper bound. The mean largest adult body masses for these taxa were estimated from our interspecific regression of mean mass and TL for adults of extant taxa (Y_(body mass, kg)_ = 5.00x_(total length, m)_^2.846^; R^2^ = 0.93). (Note: the upper bound bite-force estimate for *Deinosuchus riograndensis* is more tenuous since the largest known fossil crocodilian specimens greatly exceed the neontological size range studied here.)
